# Design and Analysis of a Low-Coupling Parallel Piezoelectric Nanopositioner Based on a Pseudo-Symmetric Structure

**DOI:** 10.3390/mi17070779

**Published:** 2026-06-26

**Authors:** Lingchen Meng, Qi Wang, Tianyi Zhang, Peng Yan

**Affiliations:** 1 Key Laboratory of High-Efficiency and Clean Mechanical Manufacture of MOE, School of Mechanical Engineering, Shandong University, Jinan 250061, China; mlc_sdu@foxmail.com (L.M.); wangqi2023@163.com (Q.W.); 2 China Center for Information Industry Development, Beijing 100846, China

**Keywords:** nanopositioning stage, compliant mechanism, displacement amplifier, decoupling mechanism

## Abstract

To meet the increasing demands for large stroke and low cross-axis coupling in precision instruments such as atomic force microscopy (AFM), a low-coupling parallel piezoelectric nanopositioning stage based on a pseudo-symmetric guiding mechanism is proposed. By integrating a compact flexure-based lever amplification mechanism with a parallel pseudo-symmetric guiding structure, the design achieves effective suppression of cross-axis coupling while maintaining a relatively large motion range. A static model is established based on Castigliano’s second theorem, and electromechanical coupled finite element analysis is performed to evaluate the output characteristics and dynamic behavior. A prototype is fabricated and experimentally validated. The results demonstrate that the stage achieves a travel range of 121 μm × 122 μm, a cross-axis coupling error ratio of 1.1%, resolutions of 7 nm and 5 nm along the X- and Y-axes, respectively, and a first natural frequency of 476 Hz. The proposed design provides a feasible approach for achieving a balance among large stroke, low coupling, and high dynamic performance in piezoelectric nanopositioning systems.

## 1. Introduction

High-precision nanopositioning systems are essential enabling technologies in a wide range of applications, including precision metrology, biomedical micromanipulation, and semiconductor inspection [1,2,3]. With the increasing size of target samples and the growing demand for high-throughput operation, next-generation systems require nanopositioning stages that simultaneously achieve nanometer-level accuracy, large travel range, high bandwidth, and effective motion decoupling [4]. Atomic force microscopy (AFM), as a representative application, imposes particularly stringent requirements on these performance metrics. However, conventional piezoelectric-driven dual-axis nanopositioning stages often struggle to suppress cross-axis coupling while maintaining large stroke and high structural stiffness, thereby limiting their performance in high-speed and high-precision applications [5,6].

To achieve a large travel range, high resolution, and fast response in nanopositioning stages, various actuation approaches have been explored. Voice coil motors (VCMs) are widely adopted in large-range micropositioning systems owing to their large stroke [7,8]. For example, Shang et al. developed a two-degree-of-freedom flexure-based stage with a working range of 1.8 mm × 1.78 mm [9]. Nevertheless, the relatively low resolution and control precision of VCMs make them unsuitable for direct nanometer-scale positioning.

Piezoelectric actuation systems, characterized by nanometer-level resolution, high stiffness, and fast response, are widely adopted in high-precision nanopositioning stages [10]. However, the intrinsic strain of piezoelectric materials is typically limited to approximately 0.1%, resulting in a restricted output stroke [11,12,13,14]. To address this issue, flexure-based displacement amplification mechanisms—such as lever-type, bridge-type, multi-stage, and parallel-type mechanisms—are commonly employed [15,16,17,18]. These mechanisms not only amplify the small displacement of piezoelectric stacks but also provide frictionless, backlash-free motion with high structural integrity and positioning accuracy, making them well-suited for nanopositioning applications [19,20,21].

Various piezoelectric-driven flexure-based nanopositioning stages have been developed based on different flexure hinges and guiding mechanisms. For example, Li et al. reported a Z-shaped flexure hinge-based stage with a motion range of 17.65 μm × 15.45 μm and a step response time of 1.6 ms [22]. Polit et al. developed an XY stage driven by two piezoelectric stacks, achieving a range of 15 μm × 15 μm with a resolution of approximately 1 nm [22]. Sun et al. proposed a large-stroke XY stage with a symmetric leaf-type flexure configuration, achieving 300 μm × 300 μm motion range and resonance frequencies above 200 Hz [23].

Despite these advances, increasing the displacement amplification ratio often introduces a trade-off between travel range and dynamic performance, due to reduced stiffness and increased inertia. In dual-axis parallel configurations, the structural complexity further exacerbates cross-axis coupling, making it difficult to simultaneously achieve large stroke, high stiffness, high bandwidth, and low coupling. Therefore, developing a nanopositioning stage that can balance these performance requirements remains a key challenge.

To overcome the limitations of existing nanopositioning stages, a low-coupling dual-axis parallel piezoelectric nanopositioning stage based on a pseudo-symmetric guiding design is proposed. The design targets the suppression of cross-axis coupling induced by flexure-based amplification mechanisms. A pseudo-symmetric guiding unit is introduced to achieve stiffness decoupling between motion and coupling directions, while a reconfigured lever-based amplification mechanism enables a more compact structure without sacrificing displacement amplification. Consequently, the proposed stage achieves reduced coupling error while maintaining a large stroke and high dynamic performance.

The remainder of this paper is organized as follows. Section 2 describes the structural design and working principles. Section 3 presents the static modeling based on Castigliano’s second theorem. Section 4 provides a finite element analysis of the static and dynamic characteristics. Section 5 reports the experimental validation, followed by conclusions in Section 6.

## 2. Mechanical Design

### 2.1. Parallel Configuration Design

**Figure 1:**
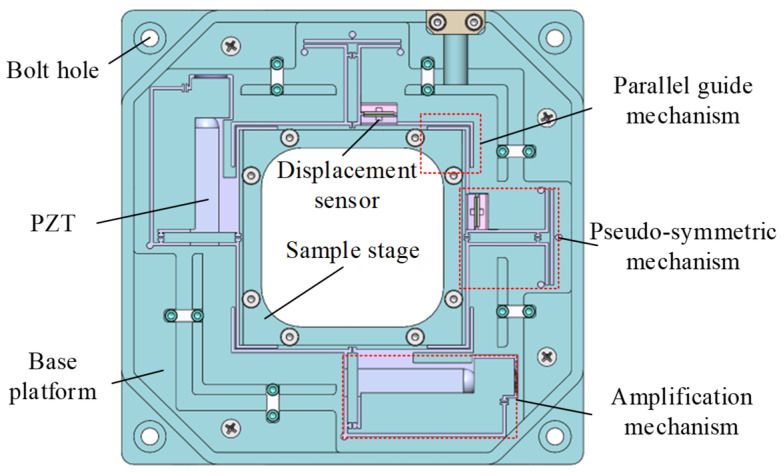
Overall layout of the proposed pseudo-symmetric parallel nanopositioning stage.

A low-coupling dual-axis parallel nanopositioning stage is developed based on a pseudo-symmetric guiding unit, as shown in Figure 1. Compared with conventional designs, the proposed structure improves stiffness decoupling by introducing pseudo-symmetric guiding units.

The stage comprises a base, lever-based flexure amplification mechanisms, a motion stage, a parallel decoupling structure, pseudo-symmetric guiding units, piezoelectric actuators, and capacitive sensors. A rotationally symmetric layout is adopted for the driving and guiding units to achieve stiffness decoupling along both axes, while two orthogonally arranged capacitive sensors enable nanometer-resolution displacement measurement.

### 2.2. Lever-Based Displacement Amplification Mechanism

**Figure 2:**
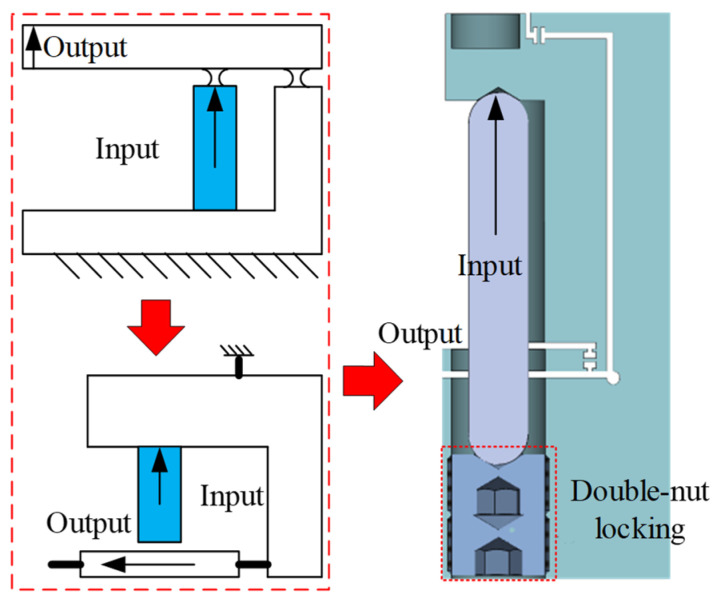
Evolution of the lever-based displacement amplification mechanism.

The limited stroke of piezoelectric actuators imposes a fundamental constraint on nanopositioning applications, motivating the development of a flexure-based displacement amplification mechanism. The evolution of its configuration is illustrated in Figure 2. Compared with conventional bridge-type, triangular-type, and lever-type amplification mechanisms, the proposed flexure lever amplifier provides enhanced output stiffness and greater structural adaptability, enabling large displacement amplification without compromising mechanical robustness.

To achieve a compact structural layout, the kinematic configuration of the conventional lever amplification mechanism is rearranged such that the input and output motions are oriented orthogonally. This arrangement reduces the overall footprint of the amplification mechanism, facilitating its integration into space-constrained assemblies. Furthermore, a decoupling flexure hinge is incorporated at the output stage to suppress the parasitic rotational motion inherently introduced by the lever mechanism, thereby improving the linearity of the amplified output motion.

From a manufacturing and operational perspective, the piezoelectric actuator is inserted into a slot in the intermediate layer of the mechanism to apply a compressive preload. A dual-screw preloading scheme further enhances the stability and reliability of the preload during dynamic operation. This design ensures consistent actuation performance and enhances the durability of the amplification system under repetitive high-frequency operation.

### 2.3. Pseudo-Symmetric Guiding Mechanism

In conventional dual-axis parallel nanopositioning stages, the guiding mechanism is typically realized using orthogonally arranged leaf-spring flexures. Due to geometric symmetry, the guiding stiffness on both sides of the moving stage is balanced, and the off-diagonal stiffness terms vanish, i.e., $k_{xy}=k_{yx}=0$, ensuring decoupled motion along the two axes.

The introduction of a displacement amplification mechanism, however, generally induces stiffness asymmetry. The lever unit adds an equivalent lateral stiffness $k_{amp}$ to the actuated branch, while the opposite branch retains the intrinsic stiffness $k_{g}$, yielding a mismatch $\Delta k=(k_{g}+k_{amp})-k_{g}=k_{amp},$ which shifts the elastic center away from the geometric center and generates non-zero coupling stiffness, causing parasitic transverse motion.

This effect arises from the inherent structural asymmetry introduced by the amplification unit rather than from manufacturing tolerances. Existing quasi-symmetric designs can partially mitigate the parasitic motion through geometric layout optimization, but they do not compensate for the additional stiffness, leaving residual coupling that limits the attainable motion decoupling of the stage.

**Figure 3:**
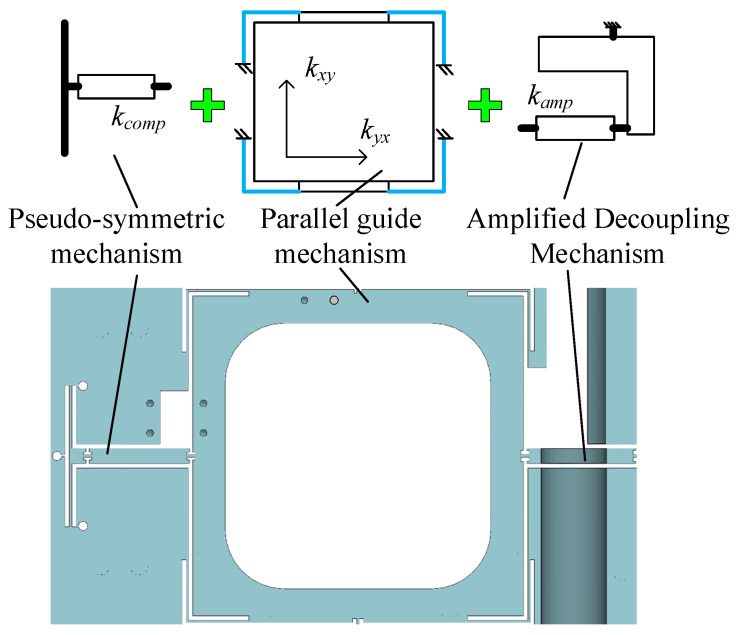
Decoupling design based on a pseudo-symmetric guiding mechanism.

A pseudo-symmetric guiding mechanism is proposed to overcome the stiffness imbalance introduced by displacement amplification units. In this configuration, the compliant guiding structure is geometrically asymmetric, yet achieves stiffness equivalence on both sides of the motion direction through a compensation flexure with stiffness $k_{comp}$ calibrated to match $k_{amp}$. This equivalence, rather than geometric symmetry, defines the pseudo-symmetric configuration and theoretically reduces $k_{xy}$ to zero. As shown in Figure 3, the compensation flexure is incorporated on the non-actuated branch of each guiding unit, with its geometry specifically designed to satisfy the stiffness-matching condition. Compared with fully symmetric stages, which maintain inherent stiffness balance but cannot accommodate amplification mechanisms without significant layout penalties, the proposed design actively compensates for stiffness asymmetry, enabling both large stroke and effective decoupling in a compact structure.

By integrating the pseudo-symmetric guiding mechanism with the lever-based amplification unit, the stage restores stiffness balance along the parasitic direction while preserving a large output stroke. The geometry and material properties of the critical flexure hinges are systematically derived from the static model presented in Section 3, providing an analytical basis for optimizing the compensation unit.

## 3. Mathematical Model

**Figure 4:**
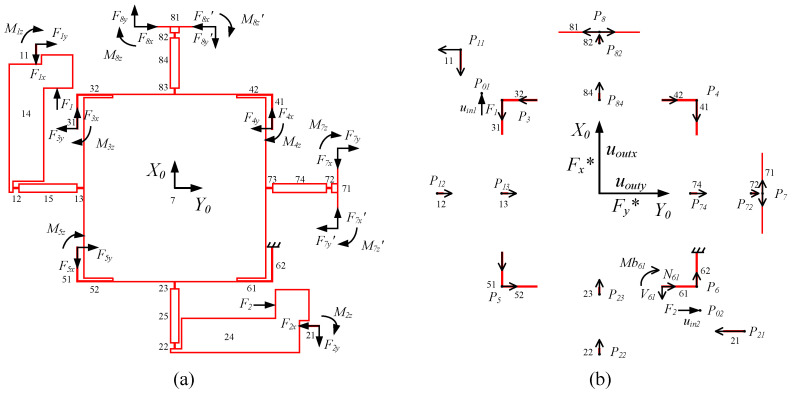
Static model of the parallel XY nanopositioning stage: (**a**) hinge constraint forces; (**b**) hinge internal forces and coordinate system.

In this section, a static model of the proposed pseudo-symmetric parallel nanopositioning stage is developed to guide the design of the critical flexure hinges. The static model of the parallel 2-DOF stage is shown in Figure 4. Under the driving force $F={\left[F_{1},F_{2}\right]}^{T}$ generated by the piezoelectric actuator, the elongation at the actuator end is defined as the input displacement of the system and denoted as $u_{in}={\left[u_{in1},u_{in2}\right]}^{T}$. The output displacement at the end of the motion stage is defined as $u_{out}={\left[u_{outx},u_{outy}\right]}^{T}$. Through static analysis, the equivalent stiffness coefficients $K_{in}\;$and $K_{out}$ are derived, satisfying:

(1) $$\left\{\begin{array}{c} F=K_{in}\cdot u_{in}\\ F=K_{out}\cdot u_{out} \end{array}\right.,$$

The stiffness matrix $K_{in}$ is a 2 × 2 symmetric matrix, where the diagonal terms $k_{in}=k_{in,1}=k_{in,2}$ represent the stiffness along the *X*- and *Y*-axes, and the off-diagonal terms $k_{xy}=k_{yx}$ denote the coupling stiffness. The former determines the travel range and the first natural frequency, while the latter governs the cross-axis coupling. Therefore, the analysis and optimization of this stiffness matrix are essential in the nanopositioning stage design.

As shown in Figure 4a, all flexure hinges are first numbered. The stage comprises eight parallel branches connecting the base and the moving stage. The first two branches serve as lever-based amplification mechanisms, while the remaining six branches provide motion along the *X*- and *Y*-axes. The sixth branch is selected as the terminal branch, and the constraint reactions at the fixed ends of the other five branches are treated as external forces:

$$R={\left[\begin{array}{ccccccccccccccc} F_{1-5x} & F_{7x} & {F_{7x}}^{\prime } & F_{8x} & {F_{8x}}^{\prime } & F_{1-5y} & F_{7y} & {F_{7y}}^{\prime } & F_{8y} & {F_{8y}}^{\prime } & M_{1-5z} & M_{7z} & {M_{7z}}^{\prime } & M_{8z} & {M_{8z}}^{\prime } \end{array}\right]}^{T}$$

Branches are numbered from the constraint ends toward the moving stage, except for the sixth branch, which follows the opposite direction. A global coordinate system X_0_Y_0_ is defined at the center of the moving stage. At each constraint, a local coordinate system is established such that the *x*-axis aligns with the flexure hinge direction (with force $F_{ix}$), the *y*-axis is determined by the right-hand rule (with force $F_{iy}$), and the out-of-plane moment is denoted as $M_{iz}$.

As shown in Figure 4b, the local coordinates of the flexure hinges in branches 1 and 2 are defined at their starting points $P_{ij}=[x_{ij},y_{ij}{]}^{T}$, whereas for branches 3–8, the coordinates are assigned at the hinge corners due to symmetry. The lengths of the hinges are defined as:

$$L={\left[\begin{array}{cccccccc} l_{11-13} & l_{21-23} & l_{31-32} & l_{41-42} & l_{51-52} & l_{61-62} & l_{71-73} & l_{81-83} \end{array}\right]}^{T}$$

Thus, the coordinates of all flexure hinges are uniquely defined in the global coordinate system *X*_0_*Y*_0_. For strain energy calculation based on Castigliano’s theorem, the internal forces of each hinge are defined as follows. Taking hinge 6–1 as an example, the axial force **N** is directed along the hinge from its starting end, the shear force **V** is perpendicular to **N**, and the bending moment **Mb** is consistent with the direction of the constraint moment.

The internal forces of each flexure hinge, including the axial force **N**, shear force **V**, and bending moment **Mb**, are obtained from the constraint reactions **R** based on local static equilibrium. By combining the compliance model of the hinges with deformation compatibility conditions, the coupled relationships among the input displacement, output displacements, and constraint reactions are established.

The total strain energy of the system is expressed as

(2) $$U=U_{a}+U_{b}+U_{v},$$

where $U_{a}$, $U_{b}$, and $U_{v}$ represent the strain energy contributions of the flexure hinges due to axial deformation, bending, and shear, respectively.

(3) $$U_{a}=\sum_{i=1}^{20}\int_{0}^{l_{i}}\frac{N{\left(i\right)}^{2}}{2ES},$$

(4) $$U_{b}=\sum_{i=1}^{20}\int_{0}^{l_{i}}\frac{Mb{\left(i\right)}^{2}}{2EI},$$

(5) $$U_{v}=\sum_{i=1}^{20}\int_{0}^{l_{i}}\frac{aV{\left(i\right)}^{2}}{2GS},$$

where *E*, *S*, *G*, and *I* represent the Young’s modulus, cross-sectional area, shear modulus, and second moment of area of the flexure hinge, respectively. Aluminum alloy 6061 is selected as the structural material. The material properties are listed in Table 1.

Since the displacements at the points where the reaction forces **R** are applied are zero, Castigliano’s theorem [24] yields

(6) $$\frac{\partial U}{\partial R_{i}}=0\;\;\;i=1,2,\ldots ,27,$$

Substituting Equation (2) into Equation (6) and considering all geometric parameters as constants yields

(7) $$A_{27\times 27}•R=aF_{1}+bF_{2},$$

(8) $$R=A^{-1}\left(aF_{1}+bF_{2}\right),$$

Equation (8) describes the linear relationship between the reaction forces *R* and the input forces $F_{1}$ and $F_{2}$. Substituting it into Equation (2) and differentiating with respect to $F_{1}$ and $F_{2}$ yields the corresponding input displacements $u_{in1}$ and $u_{in2}$, i.e.,

(9) $$\left\{\begin{array}{c} u_{in1}=\frac{\partial U}{\partial F_{1}}\\ u_{in2}=\frac{\partial U}{\partial F_{2}} \end{array}\right.\begin{array}{cc} \Rightarrow & u_{in}=C_{in}•F \end{array},$$

where ***C****_in_* represents the 2 × 2 input compliance matrix of the system. Virtual forces $F_{x}^{*}$ and $F_{y}^{*}$ are introduced at the center of the moving stage along the $X_{0}$- and $Y_{0}$-axes, respectively, as shown in Figure 4b. The strain energy $U^{*}$ is recalculated, and the output displacements are obtained by differentiating $U^{*}$ with respect to $F_{x}^{*}$ and $F_{y}^{*}$.

Following the derivation procedure of $u_{in}$, the internal forces of the hinge under the action of virtual forces are first obtained, including the axial force N*, shear force V* and bending moment Mb*.

(10) $$\left\{\begin{array}{c} N^{*}=N+\Delta N\\ V^{*}=V+\Delta V\\ Mb^{*}=Mb+\Delta Mb \end{array}\right.,$$

In addition, since the introduction of $F_{x}^{*}$ and $F_{y}^{*}$ only affects hinges 61 and 62, the first 18 rows of Δ**N**, Δ**V**, and Δ**Mb** are all zero. Moreover, it follows that

(11) $$\left\{\begin{array}{l} \Delta N_{13}=\delta N_{61}=F_{x}^{*}\\ \Delta N_{14}=\delta N_{64}=F_{y}^{*}\\ \Delta V_{13}=\delta V_{61}=-F_{y}^{*}\\ \Delta V_{14}=\delta V_{62}=F_{x}^{*}\\ \Delta Mb_{13}=\delta Mb_{61}=F_{x}^{*}•\left(0-y_{6}\right)+F_{y}^{*}•\left(x_{6}-h-0\right)+F_{y}^{*}•x\\ \Delta Mb_{14}=\delta Mb_{62}=F_{x}^{*}•\left(0-y_{6}\right)+F_{y}^{*}•\left(x_{6}-0\right)-F_{x}^{*}•x \end{array}\right.$$

Substituting Equation (11) into Equations (2)–(5), the total strain energy $U_{t}^{*}$ considering the virtual forces can be obtained. It should be noted that, since the virtual forces satisfy $F_{x}^{*}=F_{y}^{*}=0$, the constraint reaction force R remains unchanged. Therefore, the output displacement can be expressed as

(12) $$\left\{\begin{array}{c} u_{out,x}={\left.\frac{\partial U^{*}}{\partial F_{x}^{*}}\right|}_{F_{x}^{*}=0}\\ u_{out,y}={\left.\frac{\partial U^{*}}{\partial F_{y}^{*}}\right|}_{F_{y}^{*}=0} \end{array}\right.\begin{array}{cc} \Rightarrow & u_{out}=C_{out}•F \end{array},$$

The derived analytical model provides a quantitative framework for evaluating the stiffness and coupling characteristics of the proposed nanopositioning stage. In particular, the coupling behavior can be directly assessed through the off-diagonal terms of the compliance matrix, which serves as a key indicator for design optimization.

## 4. Numerical Simulations

**Figure 5:**
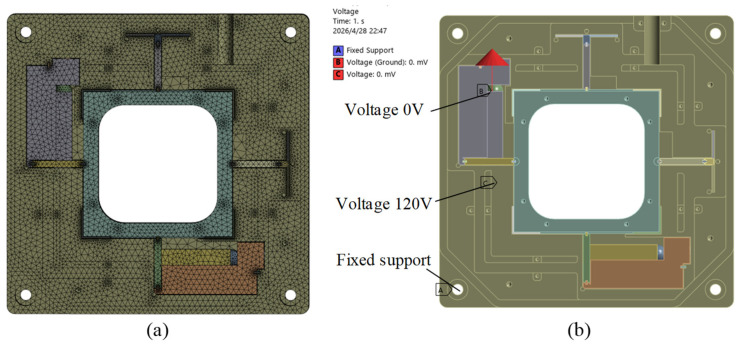
Finite element simulation settings: (**a**) mesh generation; (**b**) boundary condition settings.

A finite element analysis of the above optimized design is conducted using ANSYS Workbench 2024R2 to verify the feasibility of the proposed configuration. To ensure simulation accuracy while reducing computational cost, the geometric model established in SolidWorks 2023 is simplified by removing unnecessary manufacturing sharp corners, thereby improving mesh uniformity and quality. An adaptive meshing strategy is adopted overall. The meshing results and boundary condition settings are shown in Figure 5.

Furthermore, to investigate the relationship between the actuation force and structural stiffness of the piezoelectric drive, a piezoelectric multiphysics coupling analysis is introduced. A fixed constraint is applied to the base of the stage. The upper electrode of the piezoelectric ceramic is assigned a potential of 0 V, while the lower electrode is set to 120 V.

**Figure 6:**
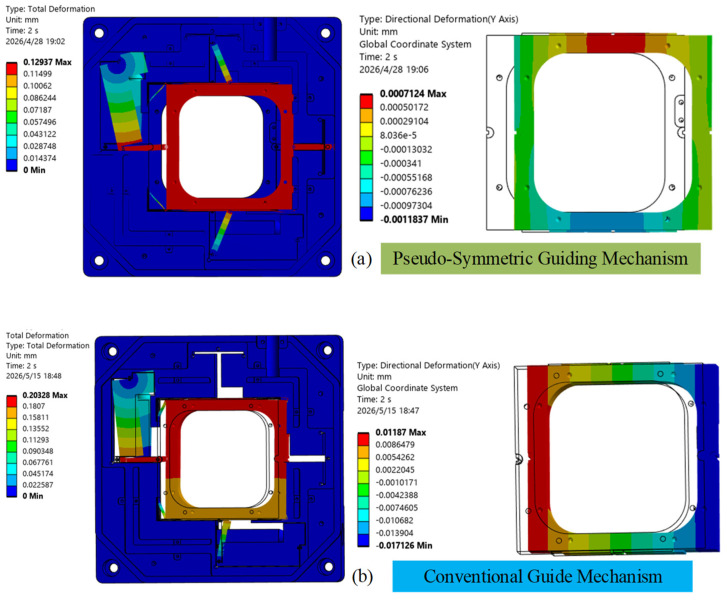
Finite element simulation results: (**a**) pseudo-symmetric guiding mechanism; (**b**) conventional guide mechanism.

The static structural simulation results are shown in Figure 6a. Taking the X-axis as an example, when the maximum input voltage reaches 120 V, the maximum output displacement in the X-direction is 129 μm, which agrees well with the theoretical prediction. Owing to the effect of the pseudo-symmetric guiding mechanism, the output displacement exhibits good linearity, and the maximum coupled displacement in the orthogonal direction is 1.1 μm (coupling ratio of 0.8%). In addition, the coupled displacement is predominantly localized at the junction between the guiding flexure hinges and the moving stage, which is largely attributed to the finite stiffness of the moving stage. To further validate the effectiveness of the proposed design, a comparative FEA study of the coupling displacement in a conventional guiding mechanism is conducted, as shown in Figure 6b. Under the same input force, the conventional mechanism exhibits a coupling ratio of 5.4%. These results demonstrate that the pseudo-symmetric design effectively suppresses cross-axis coupling displacement, confirming its advantage over conventional guiding architectures.

**Figure 7:**
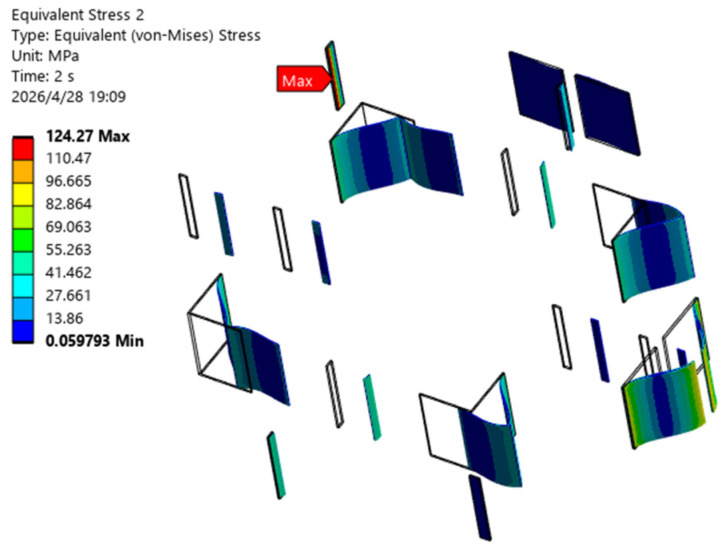
Equivalent stress verification.

Meanwhile, at the maximum output displacement, the equivalent stress distribution of the flexure mechanism is also evaluated. The results are shown in Figure 7. The stress is primarily concentrated in the flexure hinge regions, with the maximum stress occurring at the pivot flexure hinge of the amplification mechanism. The peak stress reaches 124 MPa, which is significantly lower than the allowable stress of Al 6061 alloy, thereby confirming that the structural strength meets the design requirements.

**Figure 8:**
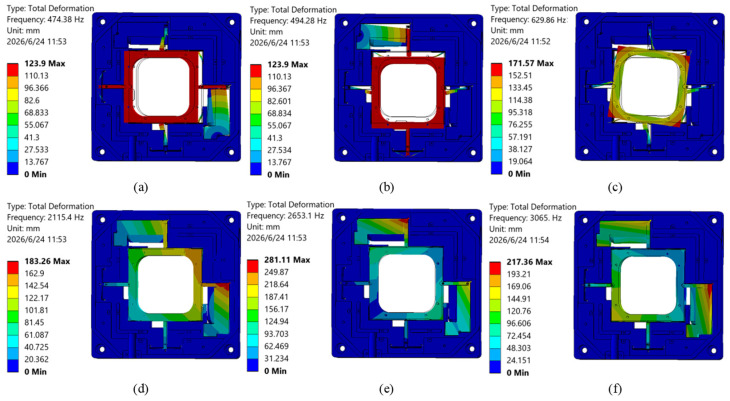
Modal analysis results: (**a**) first mode; (**b**) second mode; (**c**) third mode; (**d**) fourth mode; (**e**) fifth mode; (**f**) sixth mode.

Furthermore, a modal analysis of the proposed nanopositioning stage is performed. The first six vibration modes are shown in Figure 8. Of these, the first three modes are of primary interest: translation along the X-direction, translation along the Y-direction, and in-plane rotation.

Due to the characteristics of the parallel configuration, the first two modes correspond to the dominant translational modes, with natural frequencies of 474 Hz and 494 Hz, respectively. The close agreement between the natural frequencies in the two orthogonal directions indicates favorable dynamic symmetry and consistent motion characteristics. In contrast, the in-plane rotational mode appears at 629 Hz, which is significantly higher than the primary translational modes, indicating effective suppression of rotational coupling in the flexure mechanism. These results confirm that the proposed nanopositioning stage can operate effectively within a wide frequency band, making it well-suited for high-precision applications such as atomic force microscopy and precision metrology.

## 5. Experiments and Control

**Figure 9:**
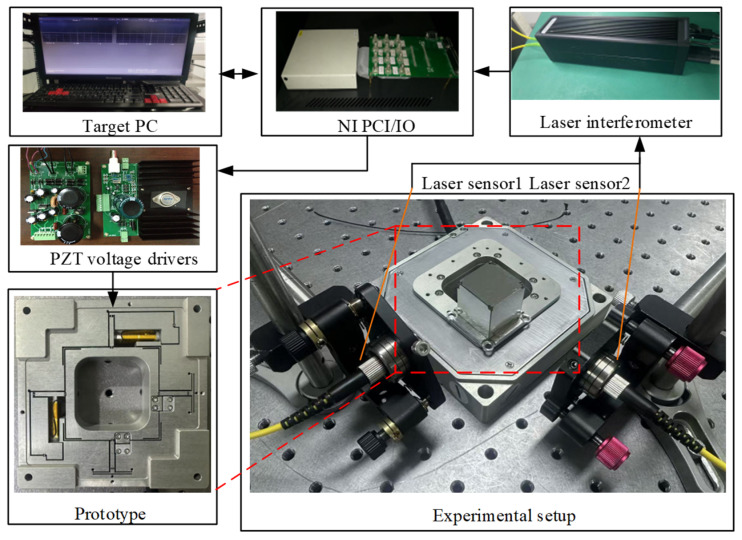
Prototype and experimental system setup.

To experimentally validate the static and dynamic characteristics of the proposed nanopositioning stage, a prototype was fabricated, and a dedicated experimental setup was constructed. As shown in Figure 9, the fabricated prototype is shown. All components are manufactured as an integrated structure using wire electrical discharge machining (WEDM) of 6061 aluminum alloy, thereby eliminating assembly-induced errors. Two piezoelectric stacks are installed in the actuator slots and preloaded using screws. The piezoelectric stacks used in this study are NAC2014-H20 (CTS Denmark A/S, formerly Noliac A/S, Ølstykke, Denmark). The key parameters are listed in Table 2.

The nanopositioning stage and laser interferometers are mounted on an air-bearing isolation platform. The laser interferometer selected is the IDS3010 (Attocube Systems AG, Haar, Germany), with a measurement range of 5 mm and a resolution of 1 nm. Two interferometers are arranged orthogonally to simultaneously measure displacements along the X- and Y-axes.

During operation, control commands generated in MATLAB/Simulink R2023b on the host computer are transmitted to the lower-level controller and then to an NI PCI-6259 (National Instruments, Austin, TX, USA) data acquisition card. The card outputs two-channel voltage signals to high-voltage amplifiers (with a 5 kHz bandwidth, sufficient for high-frequency driving), which further amplify the signals to drive the corresponding piezoelectric actuators for stepwise motion. The stage’s output displacement is measured by laser interferometers, acquired via the analog input channels of the NI PCI-6259, and stored in the lower-level controller for subsequent data processing and control optimization.

**Figure 10:**
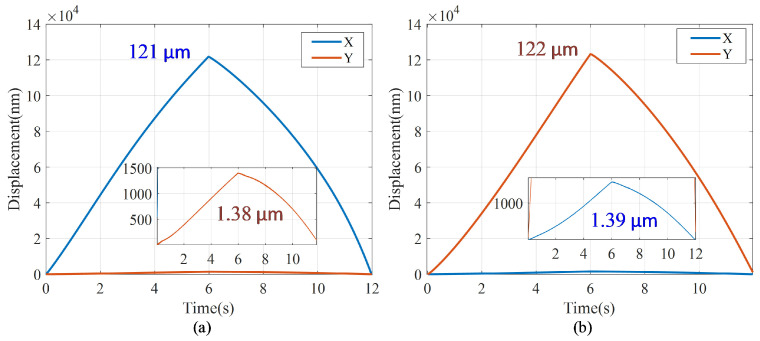
Stroke and coupled displacement measurements: (**a**) X-axis response; (**b**) Y-axis response.

The maximum stroke and cross-axis coupling of the proposed stage were characterized by applying a 120 V input to either the X- or Y-axis, while the orthogonal axis was maintained at 0 V (Figure 10). The measured travel ranges are 121 μm along the X-axis and 122 μm along the Y-axis, with corresponding cross-axis coupling displacements of 1.38 μm (1.14%) and 1.39 μm (1.13%), respectively. These results demonstrate that the pseudo-symmetric guiding mechanism effectively suppresses parasitic motion, consistent with the design objective of achieving minimal cross-axis coupling while maintaining a large output stroke.

Comparison with simulation results shows good agreement in both stroke and coupling behavior. Minor discrepancies are mainly attributed to simplifications in the boundary conditions of the numerical model and dimensional deviations arising from fabrication, which slightly affect the stiffness distribution and consequently the parasitic displacement. Notably, the obtained coupling ratios are significantly lower than those reported for conventional parallel nanopositioning stages (typically around 3%), indicating that the pseudo-symmetric guiding mechanism substantially enhances the decoupling performance of the nano-positioning system.

**Figure 11:**
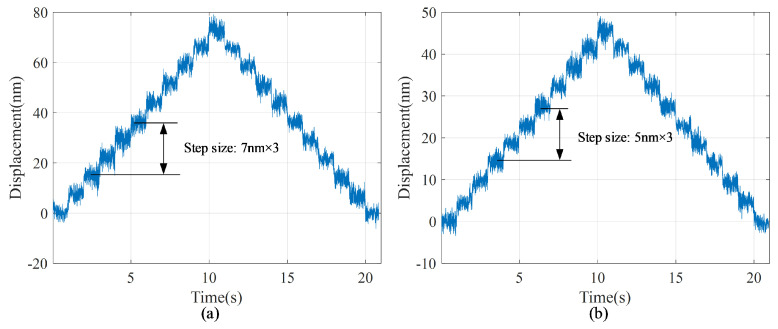
Step size characterization test: (**a**) X-axis results; (**b**) Y-axis results.

To investigate the minimum achievable incremental motion of the proposed na-nopositioning stage, a stepwise triangular voltage signal (0–60 mV) was applied to the stage. As shown in Figure 11, clear and stable nanoscale step displacements were achieved along both axes. The minimum distinguishable step sizes were approximately 7 nm and 5 nm for the X- and Y-axes, respectively, indicating the capability of the proposed stage to achieve stable nanometer-scale incremental positioning.

**Figure 12:**
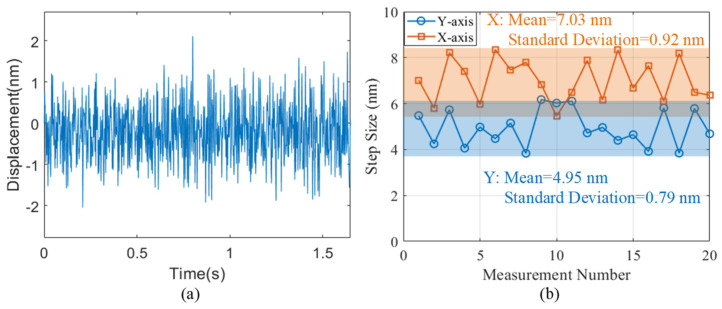
Resolution characterization: (**a**) static noise measurement of the sensing system; (**b**) analysis of step sizes under continuous step-wave excitation.

Figure 12 further presents the sensor noise floor and the statistical analysis of the stepping performance to validate the observed nanoscale motions and quantitatively determine the positioning resolution. During a 1.5 s static measurement, the positioning system exhibited an RMS noise of 0.6374 nm, corresponding to a 3σ noise floor of 1.81 nm, which is well below the measured step sizes. In addition, twenty consecutive step displacements were statistically analyzed. The average step sizes were 7.03 nm and 4.95 nm for the X- and Y-axes, with corresponding standard deviations of 0.92 nm and 0.79 nm, respectively. The close agreement between the average and observed step sizes, together with the small standard deviations, demonstrates excellent repeatability of the nanoscale incremental motions. Considering the low measurement noise and the high repeatability of the stepping performance, the positioning resolutions of the X- and Y-axes were determined to be 7 nm and 5 nm, respectively.

Although the stage adopts a pseudo-symmetric architecture, a slight difference in the measured positioning resolutions between the X- and Y-axes was observed. This discrepancy is primarily attributed to inevitable manufacturing and assembly tolerances, minor stiffness mismatches of the compliant mechanism, and performance variations among the piezoelectric actuators. In addition, the laser interferometer is sensitive to environmental disturbances, including ambient vibration, temperature fluctuations, and air refractive-index variations, while electrical noise introduced by the high-voltage amplifier and sensing electronics also contributes to minor displacement fluctuations. Collectively, these factors account for the slight difference in the measured resolutions. Nevertheless, the proposed stage consistently generates stable nanoscale incremental motions with excellent repeatability, demonstrating its reliable high-resolution positioning capability.

**Figure 13:**
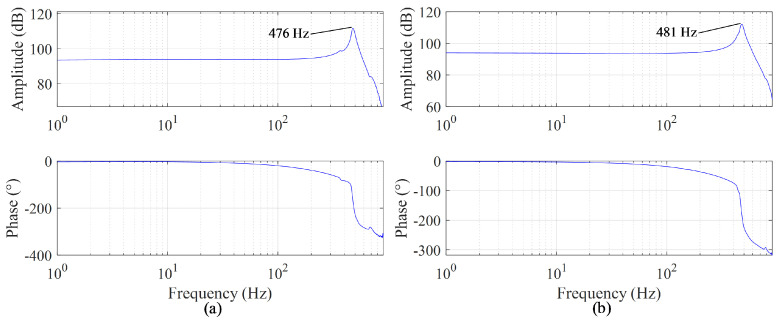
Frequency-domain response characteristics: (**a**) frequency sweep results along the X-axis; (**b**) frequency sweep results along the Y-axis.

To further validate the decoupling performance and dynamic characteristics of the proposed configuration, frequency response experiments were conducted by applying sinusoidal sweep signals to the piezoelectric actuators along the X and Y axes, respectively. The experimental results are presented in Figure 13. It can be seen that the resonant frequencies of the X and Y axes are 476 Hz and 481 Hz, respectively, which are in good agreement with the simulation results. The small discrepancies are primarily attributed to manufacturing tolerances, as well as variations in the preload of the piezoelectric stacks and contact stiffness, all of which can influence the overall stiffness of the system. Moreover, the obtained resonant frequencies satisfy the high-bandwidth requirements of applications such as atomic force microscopy (AFM) and other precision scanning systems.

A quantitative comparison between the simulation and experimental results is presented in Table 3. The results indicate that the proposed model can accurately predict the key performance characteristics of the system, and the simulation results are in good agreement with the experimental measurements, demonstrating the validity of the developed model. For the coupling ratio, a relatively larger deviation is observed compared with other performance metrics. This can be mainly attributed to the high sensitivity of cross-axis coupling to fabrication tolerances, assembly misalignments, and inevitable asymmetries in compliant structures. Even minor geometric deviations or preload variations during assembly may introduce noticeable changes in coupling behavior, leading to increased discrepancy between simulation and experimental results.

**Figure 14:**
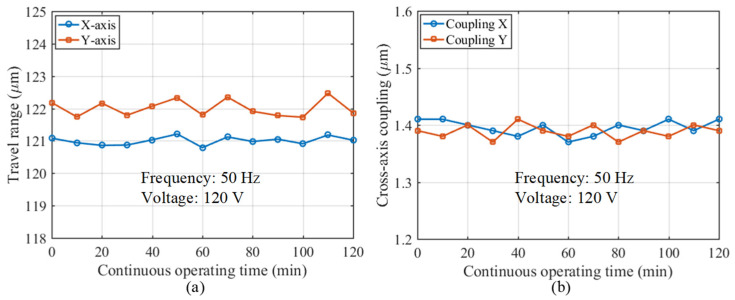
Cyclic durability test of the proposed nanopositioning stage: (**a**) travel range stability; (**b**) cross-axis coupling stability.

To further evaluate the long-term operational reliability of the proposed nanopositioning stage, a cyclic durability test was conducted under continuous operation at 50 Hz for 120 min, corresponding to approximately 3.6 × 10^5^ operating cycles. The travel ranges and cross-axis coupling displacements of both axes were measured at 10 min intervals, as shown in Figure 14. Throughout the test, the X- and Y-axis travel ranges remained close to 121 μm and 122 μm, respectively, with only minor fluctuations. Likewise, the cross-axis coupling displacements exhibited no noticeable degradation or increasing trend, indicating that the decoupling performance was well maintained. The observed variations are primarily attributed to measurement uncertainty and environmental disturbances rather than structural degradation. Overall, these results provide experimental evidence that the proposed pseudo-symmetric nanopositioning stage maintains stable positioning performance under continuous cyclic operation, supporting its operational reliability.

## 6. Discussion

The proposed pseudo-symmetric parallel nanopositioning stage achieves an effective balance between a large travel range and superior decoupling performance. The measured cross-axis coupling error of 1.1% is substantially lower than the ~3% typical of conventional parallel-kinematic stages, validating the effectiveness of the pseudo-symmetric guiding mechanism in compensating for stiffness imbalance introduced by the displacement amplification units. The experimentally measured first natural frequency (476 Hz) shows excellent agreement with FEA predictions (474 Hz), confirming sufficient dynamic stiffness for high-speed scanning applications such as atomic force microscopy.

As summarized in Table 4, the coupling ratios reported in the compared studies were all evaluated based on the full travel range of the corresponding stages, providing a consistent basis for performance comparison. The proposed stage achieves a cross-axis coupling ratio of 1.10%, representing reductions of 56.0%, 51.3%, 75.6%, and 74.4% compared with Refs. [25,26,27,28], respectively. Meanwhile, its travel range of 121 μm is comparable to the largest values reported among the listed designs and substantially exceeds those of Refs. [26,28], demonstrating that the low coupling performance is achieved without sacrificing motion range. Although the first resonant frequency of 476 Hz is lower than that of Refs. [25,26,28], it remains significantly higher than that of Ref. [27] while maintaining a considerably lower coupling ratio, reflecting a favorable balance between dynamic stiffness and decoupling performance. Furthermore, the achieved positioning resolution of 5–7 nm is substantially finer than those of Ref. [26] (31 nm) and [27] (200 nm). Overall, these results demonstrate that the proposed pseudo-symmetric architecture offers an effective design strategy for simultaneously achieving low cross-axis coupling, large travel range, and fine positioning resolution in parallel piezoelectric nanopositioning stages.

Nevertheless, several limitations remain. Out-of-plane parasitic displacements were not comprehensively characterized, and the thin flexure hinges (0.3 mm) may render high-order modal behavior sensitive to manufacturing tolerances and piezoelectric stack preload consistency. Future work will prioritize advanced control strategies for piezoelectric hysteresis compensation and the extension of the pseudo-symmetric decoupling principle to 3-DOF or 6-DOF spatial positioning systems.

## Figures and Tables

**Table 1:** Key material parameters.

Parameter Description	Value
Young’s modulus *E*	6.9 × 10^10^ N/m^2^
Shear modulus *G*	2.674 × 10^10^ N/m^2^
Width of the flexure hinge *w*	20 mm
Thickness of the flexure hinge *t*	0.3 mm
Cross-sectional area *S*	6 × 10^−6^ m^2^
Second moment of area *I*	4.5 × 10^−12^ m^4^

**Table 2:** Key parameters of the piezoelectric actuator.

Parameter	Value	Unit
Dimensions	7 × 7 × 36	mm
Stiffness	52	N/µm
Driving voltage	120	V
Free stroke	40	µm
Blocking force	2060	N
Capacitance	5060	nF

**Table 3:** Comparison between simulation and experimental results.

Parameter	Simulation (X/Y)	Experimental (X/Y)	Relative Error
Stroke (μm)	129/128	121/122	6.61%/4.92%
Coupling ratio (%)	0.8/0.9	1.1/1.1	27.27%/18.18%
Resonant Freq. (Hz)	474/494	476/481	0.42%/2.08%
Range (μm)	129/128	121/122	6.61%/4.92%

**Table 4:** Comparison of performance with existing nanopositioning stages.

Ref.	Range	Resonant Freq.	Coupling	Resolution
[25]	130 μm	586 Hz	2.50%	NA
[26]	30 μm	632 Hz	2.26%	31 nm
[27]	126 μm	94 Hz	4.50%	200 nm
[28]	17.65 μm	678 Hz	4.31%	NA
This work	121 μm	476 Hz	1.10%	5/7 nm

## Data Availability

The data that support the findings of this study are available from the corresponding authors upon reasonable request.
